# Prediction of the Spatial Origin of Puumala Virus Infections Using L Segment Sequences Derived from a Generic Screening PCR †

**DOI:** 10.3390/v11080694

**Published:** 2019-07-30

**Authors:** Sabrina Weiss, Boris Klempa, Beate Tenner, Detlev H. Kruger, Jörg Hofmann

**Affiliations:** 1 Institute of Virology, Charité – Universitätsmedizin Berlin, corporate member of Freie Universität Berlin, Humboldt-Universität zu Berlin, and Berlin Institute of Health, 10117 Berlin, Germany; 2 Institute of Virology, Biomedical Research Center, Slovak Academy of Sciences, 84505 Bratislava, Slovakia

**Keywords:** Hantavirus, Puumala virus, phylogeography, molecular epidemiology

## Abstract

To screen diagnostic specimens for the presence of hantavirus genomes or to identify new hantaviruses in nature, the pan-hanta L-PCR assay, a broadly reactive nested reverse transcription polymerase chain reaction (RT-PCR) assay targeting the L segment, is highly preferred over other assays because of its universality and high sensitivity. In contrast, the geographic allocation of Puumala virus strains to defined outbreak regions in Germany was previously done based on S segment sequences. We show that the routinely generated partial L segment sequences resulting from the pan-hanta L-PCR assay provide sufficient phylogenetic signal to inform the molecular epidemiology of the Puumala virus. Consequently, an additional S segment analysis seems no longer necessary for the identification of the spatial origin of a virus strain.

## 1. Introduction

Hantaviruses (family *Hantaviridae*) are tri-segmented negative stranded RNA viruses that are carried by small mammals (rodents, insectivores, and bats). Human infections are thought to occur through the inhalation of rodent excretions. In Germany, two pathogenic hantaviruses have been circulating, the Dobrava-Belgrade virus (DOBV), restricted to northern and eastern Germany, and Puumala virus (PUUV), endemic in the southern and western parts of the country. Most human hantavirus disease cases in Germany are caused by PUUV which usually manifests as a mild form of hemorrhagic fever with renal syndrome (HFRS), sometimes also named as nephropathia epidemica [1,2,3]. Annual case numbers vary considerably depending on the various environmental factors [4] associated with the changes in the population densities of bank voles (*Myodes glareoulus*), the PUUV reservoir hosts. Since hantavirus disease became notifiable in Germany in 2001, several outbreaks have occurred over the years with up to 2825 cases being recorded, the latest one in 2017 with 1731 notified cases [5].

Initial hantavirus disease diagnoses in Germany are based on clinical and serological data, but these do not allow for reliable typing of the virus species or even virus lineage. Reverse transcription polymerase chain reaction (RT-PCR)-based diagnostic assays are not routinely used because they are not required for case notification and virus RNA in endemic PUUV infections is detectable in patient samples only for a short period of time after onset of the first, mostly unspecific symptoms. Therefore, patient-derived PUUV genomic sequences were previously rare, and most of the PUUV genomic data was available from the reservoir hosts. Nevertheless, the recent outbreaks over the years in Germany (2007, 2010, 2012, and 2017) boosted public and medical awareness of hantaviruses, resulting in more early diagnoses and a significant build-up of PUUV genome sequence data from patients, in particular of the S segment encoding the nucleocapsid protein [6,7,8]. Combination of these sequences with the sequence data obtained from bank voles, lack of human-to-human transmission, local distribution of bank voles, and a high degree of identity between viruses of human and rodent origin allowed allocation of certain patient-derived virus sequences to defined geographic regions, thus identifying high-risk areas [7].

On the other hand, pan-hanta L-PCR assay, a broadly reactive nested RT-PCR assay, targeting the L segment [9] has recently become more widely used because of its universality and high sensitivity. Consequently, partial L segment sequences as the outcome of this screening assay are accumulating in recent years but cannot be combined with the older, S segment-based datasets [10,11].

Using sequences from the 2017 outbreak in Germany, together with human- and rodent-derived PUUV sequences from Genbank, we aimed at investigating the possible temporal and spatial changes of known geographic clusters. Furthermore, we evaluated whether the routinely generated L segment sequences provide sufficient phylogenetic signal and are suitable for studying the molecular epidemiology of the Puumala virus in Germany.

## 2. Material and Methods

As part of a nationwide alert network, serum samples from patients with acute hantavirus disease are referred to the German National Consultation Laboratory (NCL) for hantaviruses. Samples collected during 2017 until August of 2018 were tested with two different nested PCR assays, targeting the L and S segment [9,12]. Amplicons were Sanger-sequenced and processed using the MUSCLE (MUltiple Sequence Comparison by Log-Expectation) and gblocks algorithms as implemented in SeaView [13,14,15]. All sequences were analysed in the context of available sequences from previous years. A subset of samples for which both, S and L segment sequences could be generated, was analysed separately to allow for direct comparison. All maximum likelihood (ML) phylogenetic trees were inferred using the PhyML web server, including the implemented SMS (Smart Model Selection) algorithm to define the best-fit model [16,17]. The sequence analysis for the preparation of the boxplot and tanglegram, and the maps were done in RStudio using the packages ape, dendextend, and maps [18,19,20,21].

## 3. Results

During the hantavirus outbreak in 2017 in Germany and the first eight months in 2018, a total of 492 samples from patients with a clinical and serological diagnosis of hantavirus disease were referred to the NCL for molecular diagnostics and surveillance purposes. In our generic diagnostic PCR assay (addressing the L segment), 73/492 (14.8%) tested positive for hantavirus RNA. Of these, 61 (83.6%) also tested positive in a PCR targeting the S segment. All the resulting sequences were analysed in the context of respectively available sequences from previous years. Novel sequences were submitted to Genbank under accession numbers (MN026167–MN026264).

**Figure 1:**
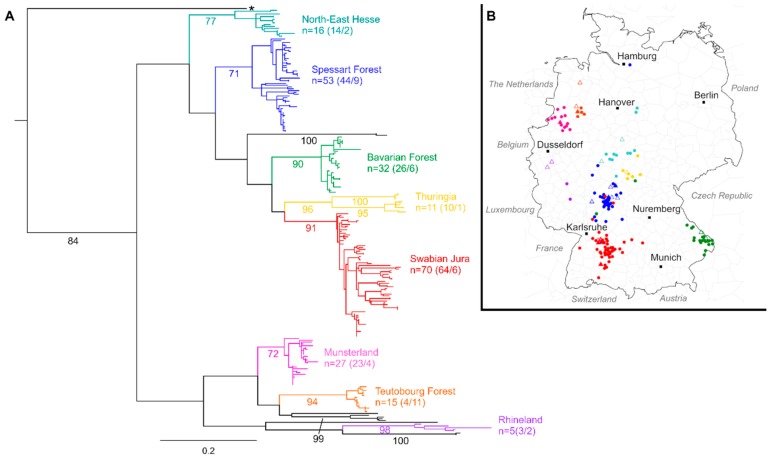
(**A**)—Phylogenetic analysis of Puumala virus S segment sequences. Human sequences stem from samples referred to the German National Hantavirus Consultation Laboratory, 2004–2018. Rodent sequences were downloaded from Genbank. A detailed list of included sequences is given in Supplementary Information S2. Maximum likelihood tree based on a 504 nt alignment, calculated using the HKY85 +G +I model of nucleotide substitution is shown. Scale bar indicates nucleotide substitutions per site. Bootstrap values (500 iterations) are given in percent for relevant clades. The tree was rooted by a human-derived sequence from Finland (accession number EU833888, not shown). Colors indicate phylogeographic clusters; black branches refer to sequences not yet assigned to any clear-defined phylogeographic cluster. n = number of sequences within the respective cluster (human origin/rodent origin). The sequence marked with a star stems from a patient with a travel history and known rodent exposure abroad. (**B**) Map showing the allocation of Puumala virus sequences in Germany. Circles indicate human-derived sequences; triangles indicate rodent-derived sequences. Multiple sequences originating from the same location are depicted only once for visibility. Colors are according to clusters in the phylogenetic tree (**A**). Black squares indicate major German cities to facilitate orientation.

When analysing PUUV S segment sequences obtained from patients during 2017 and 2018 together with sequences available in Genbank, 194/206 (94.2%) human-derived sequences fall into well-supported phylogeographic clusters (Figure 1A). The regional allocations are supported by sequences derived from bank voles captured in the respective areas, and are mostly in concordance with the putative place of infection as determined by residence or known travel history of the patients (Figure 1B and Supplementary Information S1) [5]. In each cluster, sequences from multiple years are interspersed, showing no temporal signal. For clarity, phylogeographic clusters are only colored when comprised of five or more sequences. A detailed phylogenetic tree of S segment sequences can be found in the Supplementary Figure S2.

**Figure 2:**
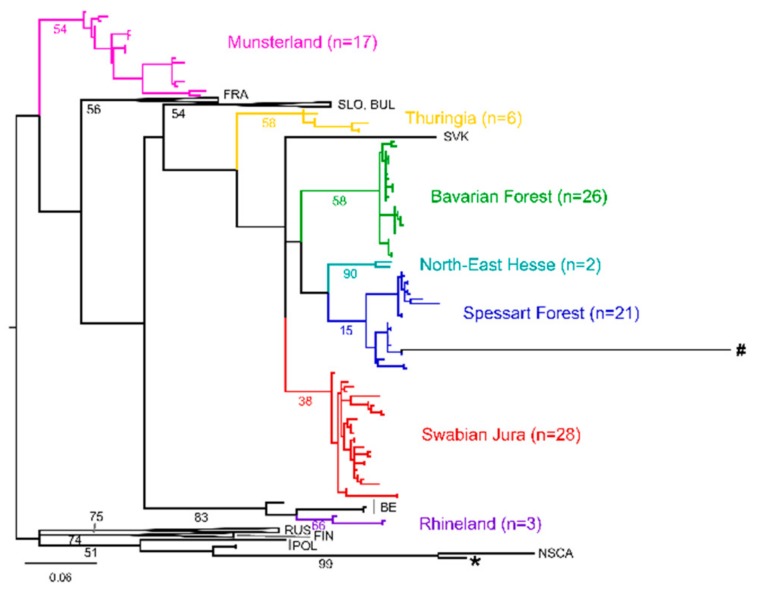
Phylogenetic analysis of Puumala virus L segment sequences. Colors indicate phylogeographic clusters in Germany. The maximum likelihood (ML) tree based on a 288 nt alignment, calculated using the GTR +G model of nucleotide substitution is shown. The tree is midpoint rooted. The scale bar indicates nucleotide substitutions per site. Bootstrap values (500 iterations) are given in percent for relevant clades. Sequences marked with a star and hash stem from patients with reported travel history abroad.

We then analyzed sequences derived from the L segment and observed a similar picture. Of all the available PUUV L sequences obtained from patients in Germany, 91/97 (93.8%) fall into well-supported phylogenetic clusters that are concordant to those seen in the S segment based analysis, without any sign of temporal clusters (Figure 2).

**Figure 3:**
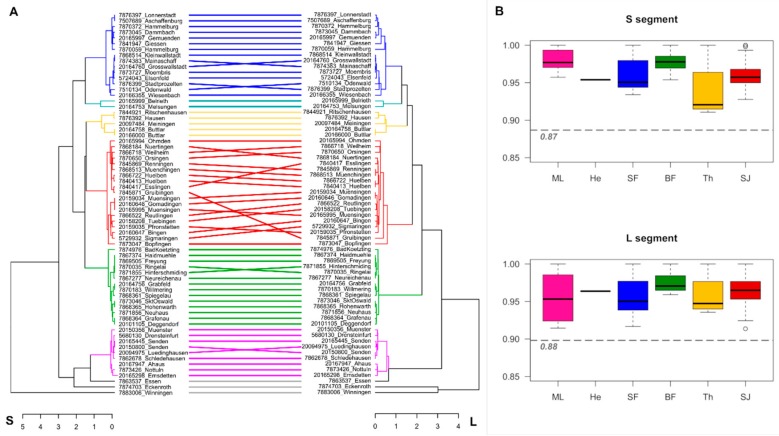
(**A**)—Tanglegram of human-derived PUUV sequences based on phylogenetic trees of the S (left side) and L (right side) segments. (**B**) Boxplots showing the pairwise identities (PWI) within defined phylogeographic clusters. Gray dashed line, and the number below indicates the mean PWI between all sequences in the dataset (87% and 88% for S and L segment alignment, respectively). Colors indicate phylogeographic regions: Pink = Muensterland, turquoise = North-East Hesse, blue = Spessart Forest, green = Bavarian Forest, yellow = Thuringia, red = Swabian Jura.

To allow for a direct comparison, we used all samples from 2015–2018 for which both sequences (L and S segment derived) were available and compared the resulting phylogenetic trees. While the overall topologies of the ML trees slightly differ, the previously identified phylogenetic and geographic clusters remain stable, as shown in Figure 3A. The pairwise identity (PWI) of sequences within those phylogeographic clusters averages to ≥93% for both, S and L segment derived sequences, whereas the average PWIs between sequences from distinct clusters are ≤88%, as illustrated in Figure 3B.

## 4. Discussion

Hantavirus disease caused by Puumala virus belongs to the most important notifiable virus diseases endemic in Germany. Analyses of patient’s sequences obtained in Germany between 2004 and 2018 revealed very low longitudinal variations within defined outbreak regions. In agreement with epidemiological data, this indicates a stable circulation of distinct PUUV variants in defined geographic areas [5].

Since multiple hantavirus species have been circulating in Germany and also imported infections have to be taken into consideration, it is highly advantageous that the diagnostic RT-PCR assay targets all so-far known hantavirus species. The L segment sequences obtained as the outcome of the pan-hanta L-PCR assay are usually used only for molecular screening and identification of the hantavirus species. For further phylogenetic studies at the species level, additional S segment sequences had been assumed to be essential. Our study indicates that this is no longer strictly necessary.

Moreover, in this study, S segment sequences were not obtained for all pan-hanta L-PCR positive samples. Consequently, relying on S segment sequences for further analysis would neglect that 16% for which only the L segment sequences are available. Here we showed that despite a very large degree of amino acid conservation (median identity between all sequences 0.98) on the L segment sequence, the nucleotide differences (Figure 3B) seem to be sufficient for meaningful phylogenetic analyses. While the tree topologies slightly differ, the main phylogeographic clusters remain identical with those identified with S segment analyses, albeit with lower statistical support. This is in accordance to data from Slovenia, where a correlation of phylogenetic and geographical clustering based on PUUV L sequences has also been described [22].

It was previously shown that sequences from viruses circulating outside Germany could be identified using data from the S segment [5]. When analyzing available patient L segment sequences, we observe two outliers within our dataset, represented by long branches compared to the rest (Figure 2; sequences marked with a star and a hash). Both patients reported travel histories abroad with possible rodent exposure. One patient (sequence marked with a star) returned from Swedish Lapland prior to developing symptoms. Accordingly, L and S segment sequences obtained from this patient cluster within the North-Scandinavian PUUV lineage (Figure 2 and Supplementary Figure S2, respectively). For one patient (marked with a hash in Figure 2), the place of infection remains elusive. The sequence does not cluster with any available sequence from Genbank, and no further information could be obtained on the reported travel destination. These results show that the L segment data is sufficient to distinguish between imported cases and the autochthonous PUUV infections in Germany.

Direct comparison of the two datasets indicates that the analysis of the L segment-based data is sufficient to allocate the patient-derived sequences to the defined phylogeographic clusters representing the endemic regions in Germany. Additional efforts to obtain the S segment sequences are not strictly necessary. Traversal lines observed upon direct comparison, and varying PWIs within clusters (Figure 3) could indicate reassortment events as previously described for PUUV [23]. However, full-length sequences would be necessary to confirm whether this is truly the case here. While short genomic segments seem sufficient for the purpose of molecular surveillance, it is always preferable to obtain sequence information of longer genomic stretches and from all genome segments. This not only enables more reliable and precise estimation of evolutionary relationships but also allows for studying the occurrence of segment reassortments and its relevance for the PUUV emergence dynamics [24].

In summary, we have shown that molecular epidemiologic studies of Puumala virus can be performed using exclusive data obtained from the routine (L segment-based) molecular diagnostic assay. This leads to a significant reduction of the time required from sample receipt to first results, which can be crucial for timely public health interventions in acute outbreak situations. Despite the recent progress in understanding PUUV epidemiological situation in Germany, continuous molecular surveillance remains a necessary tool to detect new virus variants and putative new outbreak regions and to face new challenges related with global climate and social changes.

## Supplementary Materials

The following are available online at https://www.mdpi.com/1999-4915/11/8/694/s1.
