# Supplementary figures and images for: Prediction of the Spatial Origin of Puumala Virus Infections Using L Segment Sequences Derived from a Generic Screening PCR
Source: Viruses. 2019 Jul 30;11(8):694. doi: 10.3390/v11080694 (PMC6724045; doi:10.3390/v11080694)

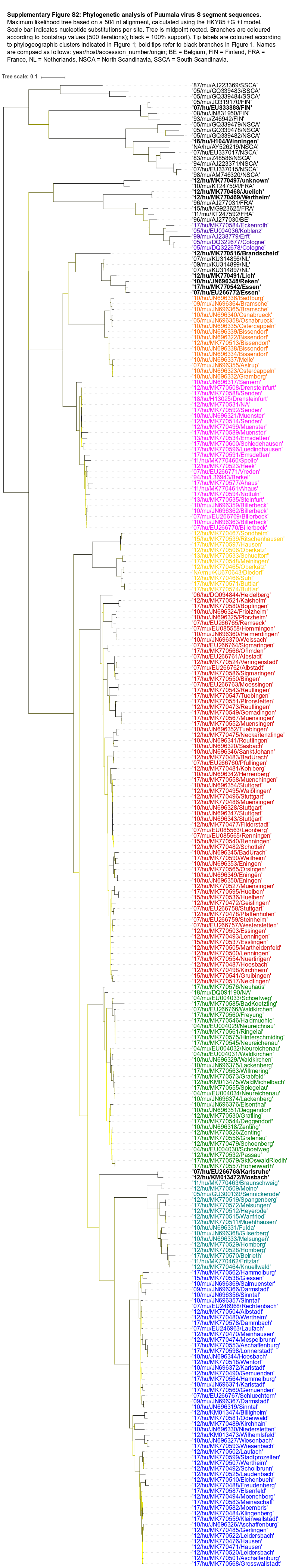

Supplement: Supplementary file 1 [file viruses-11-00694-s001.zip › S2.png]
